# Catechin and Procyanidin B_2_ Modulate the Expression of Tight Junction Proteins but Do Not Protect from Inflammation-Induced Changes in Permeability in Human Intestinal Cell Monolayers

**DOI:** 10.3390/nu11102271

**Published:** 2019-09-21

**Authors:** Massimiliano G. Bianchi, Martina Chiu, Giuseppe Taurino, Furio Brighenti, Daniele Del Rio, Pedro Mena, Ovidio Bussolati

**Affiliations:** 1 Laboratory of General Pathology, Department of Medicine and Surgery, University of Parma, 43125 Parma, Italy; massimiliano.bianchi@unipr.it (M.G.B.); martina.chiu@unipr.it (M.C.); giuseppe.taurino@studenti.unipr.it (G.T.); 2 Human Nutrition Unit, Department of Food & Drug, University of Parma, 43125 Parma, Italy; furio.brighenti@unipr.it (F.B.); pedromiguel.menaparreno@unipr.it (P.M.); 3 Human Nutrition Unit, Department of Veterinary Science, University of Parma, 43125 Parma, Italy; 4 Microbiome Research Hub, University of Parma, 43124 Parma, Italy; 5 School of Advanced Studies on Food and Nutrition, University of Parma, 43121 Parma, Italy

**Keywords:** catechin, claudin-7, flavan-3-ols, inflammation, intestinal barrier, proanthocyanidin, trans-epithelial electrical resistance (TEER), tight junctions

## Abstract

The possibility of counteracting inflammation-related barrier defects with dietary compounds such as (poly)phenols has raised much interest, but information is still scarce. We have investigated here if (+)-catechin (CAT) and procyanidin B_2_ (PB_2_), two main dietary polyphenols, protect the barrier function of intestinal cells undergoing inflammatory stress. The cell model adopted consisted of co-cultured Caco-2 and HT29-MTX cells, while inflammatory conditions were mimicked through the incubation of epithelial cells with the conditioned medium of activated macrophages (MCM). The epithelial barrier function was monitored through trans-epithelial electrical resistance (TEER), and ROS production was assessed with dichlorofluorescein, while the expression of tight-junctional proteins and signal transduction pathways were evaluated with Western blot. The results indicated that MCM produced significant oxidative stress, the activation of NF-κB and MAPK pathways, a decrease in occludin and ZO-1 expression, and an increase in claudin-7 (CL-7) expression, while TEER was markedly lowered. Neither CAT nor PB_2_ prevented oxidative stress, transduction pathways activation, ZO-1 suppression, or TEER decrease. However, PB_2_ prevented the decrease in occludin expression and both polyphenols produced a huge increase in CL-7 abundance. It is concluded that, under the conditions adopted, CAT and PB_2_ do not prevent inflammation-dependent impairment of the epithelial barrier function of intestinal cell monolayers. However, the two compounds modify the expression of tight-junctional proteins and, in particular, markedly increase the expression of CL-7. These insights add to a better understanding of the potential biological activity of these major dietary flavan-3-ols at intestinal level.

## 1. Introduction

Impairment of the intestinal barrier is involved in the pathogenesis of inflammatory bowel disease (IBD) [1]. In active forms of Crohn’s disease (CD) or ulcerative colitis (UC), the increased permeability of intestinal epithelium is likely attributable to severe epithelial damage, including enhanced apoptosis and mucosal ulceration [2]. Conversely, the effects of inflammatory cytokines on tight junctional complexes are the determinants of increased permeability in quiescent IBD and may constitute both a primary dysfunction and the factor responsible for the progression to chronic mucosal inflammation due the persistently activated macrophages [3]. 

Several components of the inflammatory milieu have been involved in permeability increase. Tumor necrosis factor alpha (TNFα) is the cytokine most often associated with epithelial tight junction (TJ) dysregulation and intestinal barrier impairment [4,5,6], but several other cytokines, such as IL-13 [7], interferon gamma (IFN-γ) [8], IL-6 [9], or IL-1beta (IL-1β) [10] have also been involved. These effects are often attributed to the down-regulation [8] or the up-regulation [7] of specific TJ proteins, possibly associated with their phosphorylation by MAPK or MLCK [6]. In other reports, cytokine detrimental effects on intestinal cells and, in particular, on TJ integrity have been attributed to oxidative stress [11,12,13] and to cytokine-dependent activation of NF-κB leading to the decrease of ZO-1 expression [14].

The identification of loss of TJ integrity as an important factor in IBD pathogenesis has prompted the research of chemo-protectors of the epithelial barrier. For example, butyrate and short-chain fatty acids have been described to increase claudin 1 transcription and barrier competence [15], while the pore-forming claudin 2 was down regulated [16]. Also, dietary polyphenols have been experimentally used in the attempt to reduce gut permeability and improve TJ function, interfering with inflammatory transduction pathways [17,18,19]. Nevertheless, potentially adverse effects, such as pro-oxidant activities, perturbations of transporters, and modulation of some phase I/II enzymes have been pointed out [20].

Flavan-3-ols are among the most representative classes of dietary (poly)phenols [21,22], which are attracting much interest, given their putative beneficial effects in the framework of gut health [23,24,25]. (+)-Catechin (CAT) is one of the main flavan-3-ol monomers in the human diet, while procyanidin dimer B_2_ (PB_2_) is a widespread dimeric proanthocyanidin. These two flavan-3-ols are present at high concentrations in cocoa and dark chocolate, apples, red wine, tea, and some stone fruits. Both kinds of flavan-3-ols promoted anti-oxidant responses in human colorectal epithelial cell lines, preventing reactive oxygen species (ROS) production and apoptotic cell death [26]. However, the effects and mechanisms behind the protective role of flavan-3-ols on the onset of IBD may be related to different pathways, as it has been recently reviewed [27]. 

Despite their many limitations, in vitro cell models still represent a useful tool to predict the effects mediated by natural compounds on several functional end points, such as epithelial barrier permeability. In particular, polarized Caco-2 cell monolayers, alone or in co-culture with goblet-like cells, have been widely used for the investigation of polyphenol adsorption by the intestinal epithelium [28,29,30]. Other studies have exploited the same cell culture system to study the effects mediated by natural compounds on gut function upon inflammatory insults [20,31].

One of the most predictive index of epithelial barrier integrity, studied in vitro, is the Trans-Epithelial Electric Resistance (TEER). This parameter is related to the expression of TJ proteins and, more importantly, to their distribution in polarized epithelial cells. Alteration of TEER can be experimentally induced in intestinal cell monolayers through the addition of inflammatory cytokines, used individually or as a mixture, or of other inflammatory stimuli to the basolateral side of the cell system [32]. This approach has been successfully used to document the protective effects of phyto-compounds on epithelial integrity under inflammatory conditions [31,33]. In the present contribution, we have used a similar approach to investigate if CAT or PB_2_ pretreatment mitigates the impairment of intestinal barrier function due to inflammatory stimuli.

## 2. Materials and Methods 

### 2.1. Chemicals and Reagents

All chemicals were of analytical grade. CAT, PB_2_, and dimethyl sulfoxide (DMSO) were purchased from Sigma-Aldrich (St. Louis, MO, USA). Culture media and fetal bovine serum (FBS) were from Euroclone (Pero, MI, Italy).

### 2.2. Cell Culture and Experimental Treatments

Caco-2 cells, derived from human colorectal carcinoma, were purchased from ATCC, while HT29-MTX, a human colon carcinoma-derived mucin-secreting goblet cell line, were kindly provided by prof. Antonietta Baldi, University of Milan. Caco-2 cells were cultured in Minimum Essential Medium (MEM), while HT29-MTX cells were maintained in Dulbecco’s Modified Eagle’s Medium (DMEM) with high glucose (4.5 g/L) and 10 mM of sodium pyruvate. Both media were supplemented with 10% of FBS, 2 mM of glutamine and antibiotics (streptomycin 100 g/mL penicillin, 100 U/mL). Before and during treatments, all cultures were maintained in a humidified atmosphere of 5% CO_2_ in air in 10-cm dishes and passaged three times a week. 

For the experiments, co-cultures of Caco-2 and HT29-MTX cells were employed [34]. A mixed suspension of Caco-2 and HT29-MTX cells (7:3) was seeded in DMEM + FBS at a density of 10 × 10^4^ cells/cm^2^ into cell culture inserts with membrane filters (pore size 0.4 µm) for Falcon 24-well-multitrays (Cat. N◦3095, Becton, Dickinson & Company, Franklin Lakes, NJ, USA), and grown for 21 d until a tight monolayer was formed (TEER > 600 Ω × cm^2^) with a medium replacement every three days.

Stock solutions of PB_2_ and CAT (20 mM in DMSO) were diluted in culture medium in order to obtain two preparations of each compound at the concentration of 250 µM, which were then added to the apical side of the culture system at the concentration of 50 µM. After 24 h of pre-incubation, the basolateral culture medium was substituted with conditioned (see below) or not conditioned medium, as specified for each experiment. At the end of the experiment, both apical and basolateral media were stored at −20 °C for further analysis. 

### 2.3. Medium Conditioning by Raw264.7 Macrophages

Murine peritoneal macrophages Raw264.7, obtained from the Istituto Zooprofilattico della Lombardia e dell’Emilia Romagna (Brescia, Italy), were grown in DMEM completed with FBS 10%, streptomycin (100 μg/mL)/penicillin (100 U/mL) and glutamine (4 mM) in 10-cm dishes in standard culture condition. For conditioning [35], Raw264.7 cells seeded at a density of 2 × 10^6^ cells/dish and treated with *E. coli* (strain 055:B5) lipopolysaccharide (LPS; 10 ng/mL). After 48 h, medium was collected, centrifuged at 1500× *g* for 5 min, filtered with a 0.2 μm pore-size filter, and conserved at −20 °C. This medium was defined Macrophage Conditioned Medium (MCM). Medium of macrophages not treated with LPS was processed in parallel and used as control.

### 2.4. TEER Measurement

TEER was measured using an epithelial voltmeter (EVOM, World Precision Instruments Inc., Sarasota, FL, USA). The integrity of cell monolayers was assessed just before the addition of CAT and PB_2_ (T0) and monitored every 24 h thereafter till the end of the experiments. Changes in TEER were calculated as the percentage of the initial value and normalized for the changes recorded on control cells. The equation used (Equation (1), [36]) was

(1) $$\mathrm{TEER}\;\left(\%\right)=\frac{{\mathrm{Final}\;\mathrm{TEER}}_{\mathrm{treated}}}{{\mathrm{Final}\;\mathrm{TEER}}_{\mathrm{control}}}\times \frac{{\mathrm{Initial}\;\mathrm{TEER}}_{\mathrm{control}}}{{\mathrm{Initial}\;\mathrm{TEER}}_{\mathrm{treated}}}.$$

### 2.5. Western Blot

The analyses were performed on the monolayers used for TEER measurements, modifying the method described by Rotoli et al. [37]. Briefly, the monolayers were rinsed two times with ice-cold PBS and then covered with 70 µL of Lysis buffer (20 mM Tris–HCl, pH 7.5, 150 mM NaCl, 1 mM EDTA, 1 mM EGTA, 1% Triton, 2.5 mM sodium pyrophosphate, 1 mM β-glycerophosphate, 1 mM Na_3_VO_4_, 1 mM NaF, 2 mM imidazole) supplemented with a protease inhibitor cocktail (Complete, Mini, EDTA-free, Roche, Monza, Italy). Total cell lysates were collected in Eppendorf tubes, sonicated and centrifuged at 14,000× *g* for 10 min at 4 °C to eliminate cell debris. The supernatants were then transferred in new tubes and mixed with a proportional volume of Sample buffer 4X (250 mM Tris–HCl, pH 6.8, 8% SDS, 40% glycerol, and 0.4 M DTT) before being boiled for 10 min. Samples were then loaded on 10% SDS-polyacrylamide gel, and proteins were separated for 1 h. Proteins were then blotted on PVDF membrane (Immobilon-P, Millipore, Millipore Merck Corporation, Burlington, MA, USA) for 1 h, incubated in TBS with 10% blocking solution (Western Blocking Reagent, Roche) for 1 h and exposed overnight to primary antibodies (see Table 1) diluted 1:1000 in the same solution. After three washes of 10 min each in TBS 1% Tween, membranes were exposed to the HRP-conjugated secondary antibodies in blocking solution for 1 h at RT. Visualization of protein bands was performed using Immobilon Western Chemiluminescent HRP Substrate (Millipore).

### 2.6. Immunofluorescence

The monolayers exploited for TEER measurements were visualized in confocal microscopy [38]. After the experimental treatment, cell monolayers, kept on membrane filters, were washed twice in ice-cold PBS and incubated for 5 min in absolute methanol at −20 °C. Fixed cells were then treated for 10 min with 10% of Triton-100 in PBS and incubated for further 2 h in a solution of 10% BSA and 2% of Normal Goat Serum (DAKO SpA, Milan, Italy) to block aspecific binding sites. Cell monolayers were then incubated overnight in the presence of primary antibodies (Table 1) antibodies in 10% BSA in PBS. The day after, cells were rinsed three times with PBS and exposed to the secondary antibodies Alexa Fluor 488 goat anti-mouse and Alexa Fluor 543 goat anti-rabbit for the detection of ZO-1 and claudin-7, respectively. At the end of the incubation, filters were mounted on glass slides, covered with anti-fade mounting medium to preserve fluorescence and sealed with coverslides. Immunostained cells were observed with an inverted LSM 510 Meta confocal system (Carl Zeiss, Jena, Germany) using a 40× (1.3 NA) oil objective. Single-plane confocal images were taken with excitation at at 543 nm and emission recorded through a 580- to 630-nm band pass barrier filter for Alexa Fluor 543 to visualize claudin-7; excitation at 488 nm and emission through a 515- to 540-nm band pass filter for Alexa Fluor 488 to visualize ZO-1.

### 2.7. Oxidative Stress

To prove possible anti-oxidant effects of CAT and PB2, we measured ROS production through changes in fluorescence of 2,7-di-chlorofluorescein (CM-H_2_DCFDA). Monocultures of Caco-2 and HT29-MTX cells were seeded in a 96-well plate at 20,000 cells/well. The day after seeding, CAT and PB2 were added to the culture medium at the concentration of 50 µM. After 24 h, growth medium was removed from cells and cells were incubated in pre-warmed HBSS (Hank’s balanced salt solution) containing the probe (10 µM) for 1 h at 37 °C. The loading buffer was removed and substituted with the experimental medium. 2,3,4-trihydroxybenzophenone (THB, 200 µM) was used for positive control. Fluorescence was measured every hour using as microplate reader EnSpire (Perkin-Elmer, Waltham, MA, USA) at 492–495 (ex.) and 517–527 nm (em.).

### 2.8. Statistics

Data are expressed as the mean ± SD. Statistical significance was determined by *t*-test for unpaired data. GraphPad Prism^®^ software version 6.00 (GraphPad Software Inc., San Diego, CA, USA) was used. Results were considered significant at *p* < 0.05.

## 3. Results

### 3.1. Neither CAT nor PB2 Prevent the Perturbation of Epithelial Barrier Function Induced by MCM

**Figure 1:**
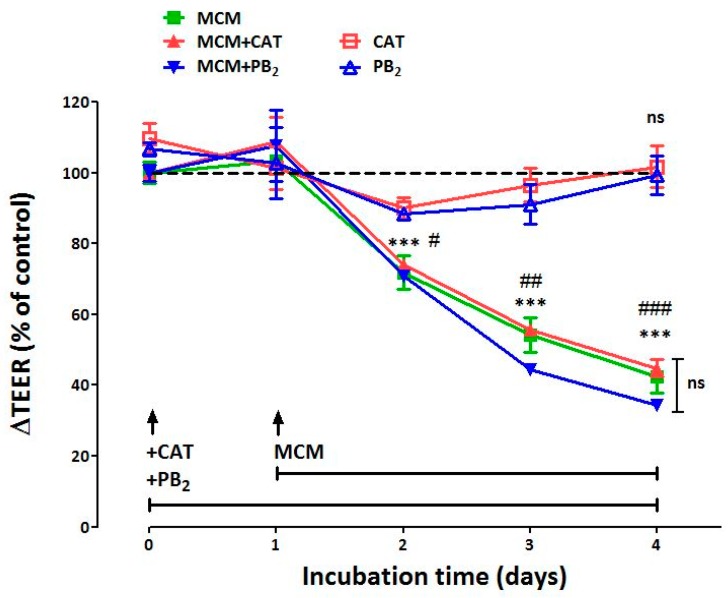
Trans-epithelial electrical resistance of Caco-2/HT29-MTX monolayers. Effect of macrophage-conditioned medium. After a tight monolayer had formed, (+)-catechin (CAT) and procyanidin B_2_ (PB_2_) (50 µM) were added, where indicated, to the apical chamber (T0). After 24 h, the medium in the basolateral chamber was replaced by conditioned medium of Raw264.7 macrophages stimulated by LPS (conditioned medium of activated macrophages (MCM), see Methods) and the incubation prolonged for further 72 h. Trans-epithelial electrical resistance (TEER) was determined, as described under Methods, at the indicated times and expressed as changes relative to control cells, calculated according to Equation 1 (see Methods). Control cells were monolayers treated with conditioned medium obtained from Raw264.7 cultures not activated by LPS (see Methods). Data are means ± SD of 3 independent determinations. *** *p* < 0.001 vs. control cultures; #, ##, ### *p* < 0.05, 0.01, 0.001 vs. monolayers treated with CAT and PB_2_ but not with MCM. The experiment was performed twice with comparable results.

Figure 1 reports the values of TEER recorded in Caco-2/HT29-MTX co-cultures, pre-incubated for 24 h in the presence of CAT or PB_2_ 50 µM and then challenged for further 72 h with MCM. TEER was measured every 24 h starting from the addition of the two compounds (incubation time 0) in order to monitor changes in monolayer permeability. The results obtained showed a clear-cut reduction (−25%) of TEER already after 24 h of exposure to MCM, compared with monolayers incubated with conditioned medium from macrophage cultures not treated with LPS. The effect was not detectable when epithelial monolayers were directly exposed to LPS (10 ng/mL, results not shown). After exposure to MCM, monolayer resistance continued to fall to reach a value 75% lower than control after 72 h of incubation. Neither CAT nor PB_2_ prevented the TEER decrease in MCM-treated cells. 

### 3.2. CAT and PB2 Differentially Affect TJ Protein Expression in MCM-Treated Monolayers

**Figure 2:**
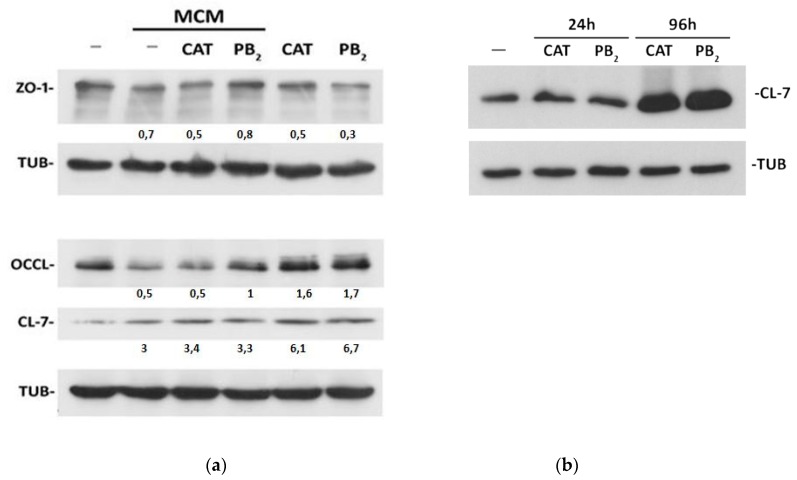
Expression of tight-junctions proteins in Caco-2/HT29-MTX monolayers. Cell monolayers were incubated for 24 h in plain growth medium or with CAT or PB_2_ (50 µM), added at the apical side. (**a**) The basolateral medium was then replaced with MCM or with the medium of Raw264.7 cultures not treated with LPS (See Methods), and the incubation prolonged for further 72 h. Protein were extracted and the expression of ZO-1, occludin and claudin-7 was determined. (**b**) Proteins were extracted at the end of the 24 h-incubation or after further 72 h of incubation. The expression of claudin-7 was determined. For both a and b, tubulin was used for loading control. The figure reports the results of representative experiments performed twice with comparable results.

The Western blot of TJ proteins, performed on the same monolayers used for TEER measurements, indicated that MCM produced a moderate decrease in ZO-1 and occludin expression, while claudin-7 (CL-7) was markedly increased (Figure 2a). A 24 h-preincubation with CAT did not substantially modify these changes, while PB_2_ prevented the decrease in the expression of occludin but not the changes in ZO-1 or CL-7 expression. When CAT and PB_2_ were used alone, without MCM treatment, they had different effects on TJ protein expression. ZO-1 expression was reduced by PB_2_ and, less evidently, by CAT. On the contrary, both polyphenols increased the expression of occludin and, more markedly, of CL-7. The effect of the two polyphenols on CL-7 required a prolonged incubation, since it was not yet detectable after 24 h of treatment (Figure 2b).

### 3.3. CAT and PB2 Partially Prevent the TJ Protein Redistribution Induced by MCM

**Figure 3:**
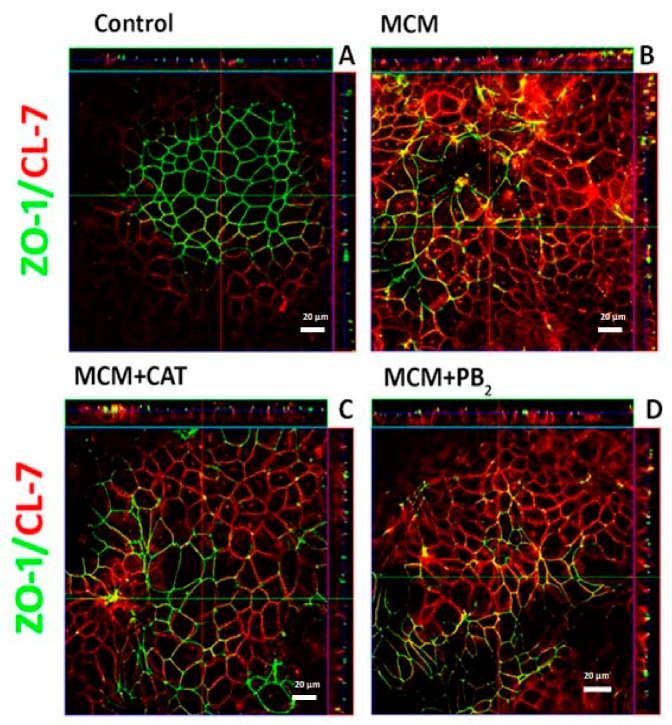
Distribution of ZO-1 and CL-7 in Caco-2/HT29-MTX monolayers. Cell monolayers were incubated for 24 h in plain growth medium or with CAT or PB_2_ (50 µM), added at the apical side. The basolateral medium was then replaced with MCM or with the medium of Raw264.7 cultures not treated with LPS (control), and the incubation prolonged for further 72 h. At the end of the incubation, cell monolayers were fixed and immunostained for claudin-7 (red) and ZO-1 (green). For each condition, a single horizontal confocal section of a representative field is shown, with orthogonal projections. The experiment has been performed twice with similar results. Bar = 20 μm.

Literature results indicate that pro-inflammatory stimuli not only affect TJ protein expression, but also their distribution in polarized monolayers, a change that has been associated with the impairment of epithelial barrier function [39,40,41]. To assess if MCM, CAT and PB_2_ were able to modify TJ protein distribution, we evaluated ZO-1 and CL-7 with confocal microscopy (Figure 3) under the same experimental conditions adopted in Figure 1.

In control monolayers, the single confocal section seems to indicate a prevalent ZO-1 expression in the center of the field with a more evident CL-7 signal at the periphery. However, as demonstrated by the orthogonal sections and by the three-dimensional reconstructions (Figure S1), the expression of the two proteins is detectable in the whole cell population. This apparent contrast reflects the imperfect alignment of the filter, on which the cells had grown, on the coverslide. Orthogonal sections clearly indicate that ZO-1 was confined to the peri-apical belt in both control and MCM-treated monolayers. The expression of the protein was decreased in MCM-treated monolayers, either in absence or in presence of CAT or PB_2_. In control monolayers, CL-7 expression pointed to a prevalent membrane distribution of the protein that involved the whole baso-lateral compartment, with very limited co-localization with ZO-1 signal. MCM-treated cells showed overexpression and altered distribution of CL-7, which exhibited clear-cut areas of co-localization with ZO-1 and was not mainly confined to the plasma membrane anymore, but seemed, instead, distributed in cytoplasmic clusters. Pre-treatment with either polyphenol mitigated CL-7 redistribution reducing the co-localization with ZO-1. However, an increase in CL-7 expression was still clearly detectable, as confirmed also by the three-dimensional reconstructions reported in Figure S1. 

### 3.4. CAT and PB2 Do Not Block the Transduction of Pro-Inflammatory Signaling

To assess if MCM activates the inflammatory response in Caco2/HT29-MTX co-cultures, we evaluated the activation state of p38 and ERK1/2 MAPK kinases. These two kinases, also involved in apoptosis and autophagy, are rapidly phosphorylated in Caco-2 cells when challenged with inflammatory cytokines [6]. The activation of the master transcription factor NF-κB has also been evaluated. Both MAPK and NFκB activation have been involved in inflammation-dependent increase of epithelial permeability [42]. 

**Figure 4:**
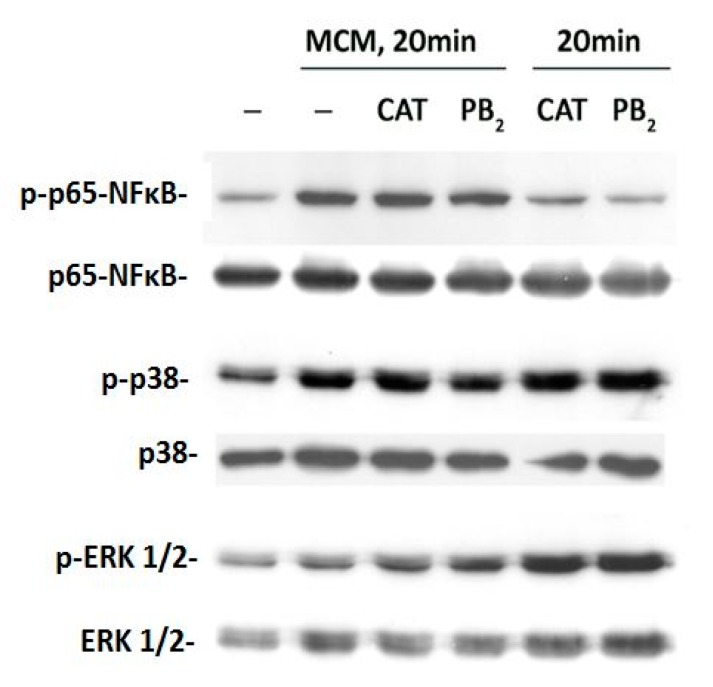
Transduction pathways activated by MCM in Caco-2/HT29-MTX co-cultures. Cell monolayers were incubated for 24 h in plain growth medium or with CAT or PB2 (50 µM). The basolateral medium was then replaced with MCM, or with the medium of Raw264.7 cultures not treated with LPS, and the incubation prolonged for further 20 min. Proteins were then extracted, and the phosphorylation of p38, ERK1/2, and NFκB was determined with Western blot. The figure reports the results of a representative experiment performed three times with qualitatively comparable results.

Results, presented in Figure 4, showed that, compared with control, MCM treatment induced a clear phosphorylation of NF-κB in cell monolayers, which was already evident after 20 min of exposure. Neither CAT nor PB_2_ pre-incubation prevented MCM-dependent activation of NF-κB. Treatment with both compounds used alone did not affect NF-κB phosphorylation. p38 was also phosphorylated in MCM-treated cells, and, also in this case, PB_2_ or CAT pre-incubation did not prevent its activation. MCM also activated the ERK1/2 branch, a change that was not attenuated, but rather augmented, by CAT and, more evidently, by PB_2_ pre-incubation. Interestingly, when CAT and PB_2_ alone were added, there was an evident, rapid increase in ERK1/2 and p38 phosphorylation compared to control.

### 3.5. Neither CAT nor PB2 Prevent MCM Dependent Production of ROS in Caco-2 and HT29-MTX Cells 

**Figure 5:**
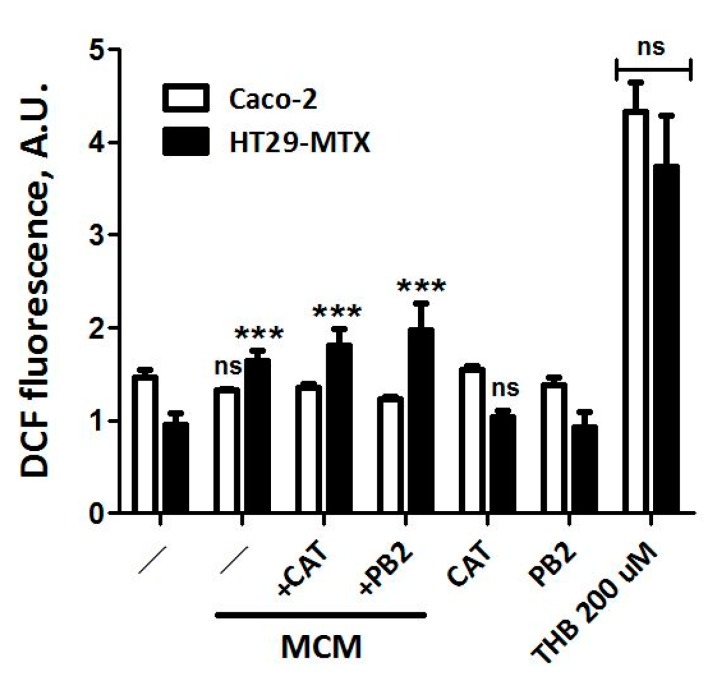
ROS production in Caco-2 and HT29-MTX cells: Effect of MCM. The day after seeding, CAT and PB2 were added to the culture medium at a concentration of 50 µM. After 24 h, medium was removed and cells were incubated for 1 h at 37°C. in pre-warmed Hank’s balanced salt solution (HBSS), containing CM-H2DCFDA (10 µM). The loading buffer was removed and substituted with control medium or MCM, in the absence or in the presence of CAT or PB_2_, as indicated. THB (200 µM) was used for positive control. Data are means of four independent determinations with SD shown. *** *p* < 0.001 vs. control, untreated culture of relative Caco-2 or HT29-MTX cells.

It is well known that inflammatory conditions promote the production of Reactive Oxygen Species in several cell models, including intestinal epithelial lines [43]. To assess if the cell types used in this study undergo oxidative stress when treated with MCM and if, in this case, CAT and PB_2_ show any protective effect, we evaluated ROS production in monocultures of Caco-2 and HT29-MTX cells exposed to MCM (Figure 5). 

After 24 h of incubation in MCM, a significant increase of ROS production was observed in HT29-MTX but not in Caco-2 cells. Neither CAT nor PB_2_ prevented the MCM dependent oxidative stress in HT29-MTX cells. In both cell lines, neither compound significantly modified ROS production under basal conditions. The oxidant compound THB, used as a nonspecific oxidative stress inducer, produced a comparable increase in ROS in the two cell types. 

These results may suggest that HT29-MTX, rather than Caco-2 cells, are the target of MCM, although it should be recalled that these experiments have been performed on monocultures grown on plastic dishes, i.e., under conditions markedly different from those employed for TEER determinations or expression studies.

## 4. Discussion

Aim of this study was to investigate if CAT and PB_2_, representatives of the most common class of dietary polyphenols, were able to protect intestinal epithelial barrier integrity under inflammatory conditions. To address this issue, we adopted an in vitro system consisting of Caco2/HT29-MTX co-culture challenged with conditioned medium (MCM) of Raw264.7 macrophages treated with LPS. Our study shows that neither CAT nor PB_2_ were capable of preventing the loss of epithelial barrier function mediated by MCM, as assessed from changes in trans-epithelial electrical resistance, even though the two polyphenols showed some capacity to modulate TJ protein expression. The apparent inefficacy of the two molecules in preserving TEER is consistent with their inability to prevent NF-κB, p38 or ERK 1/2 activation observed in the first moments of incubation with MCM. In human intestinal cells, both p38 and NF-κB can be activated by oxidative stress [44]. Indeed, HT29-MTX, but not Caco-2 cells, underwent significant oxidative stress upon incubation with MCM. Also in this case, however, CAT and PB_2_ had no significant protective effect, since ROS production was not modulated by their presence. 

The negative results obtained in this work seem in disagreement with several previous reports in which polyphenols were reported to protect the integrity of the intestinal barrier [45,46,47,48]. However, some important differences between previously published studies and this contribution should be highlighted. First, the model used in this report is different from those more commonly adopted in studies on perturbed intestinal permeability in vitro, which usually consist of monocultures of Caco-2 [49,50,51] or other cell lines [52,53,54]. Compared with those models, the co-culture system exploited here [34], based on absorbing (Caco-2) and mucus secreting (HT29-MTX) cells, is closer to the in vivo situation, since the epithelial cell monolayer is covered by a mucus layer. On the other hand, mucus can lower the amount of CAT and PB_2_ that effectively interacts with the cell monolayer and modify the response of the epithelial layer to noxious stimuli. Polyphenols have been described to protect intestinal barrier also in co-culture systems [20,31], but TEER was not considered among the parameters tested in those works. Another peculiar feature of our study is the use of chemically pure compounds used alone and not as a component of natural or artificial mixtures or extracts, which are more often exploited to assess the effects of these compounds [55,56]. Although exposure to pure polyphenols rarely occurs in vivo, the approach adopted here has the advantage to allow a clear attribution of the observed effects to the tested compounds. In addition, concentrations among studies vary substantially. Here, we used 50 μM since this concentration is line with the amount of several flavan-3-ols found in the ileal fluid of ileostomy subjects upon consumption of dietary amounts of flavan-3-ols [57,58]. Finally, the experimental condition adopted here to mimic inflammation is based on a conditioned medium of activated macrophages, which is a mixture of inflammatory factors, and not on selected cytokines. We are aware that our approach does not allow us to discriminate easily what the actual stimuli that may cause epithelial alteration is, but this condition is certainly closer to what occurs during inflammatory conditions in vivo.

**Figure 6:**
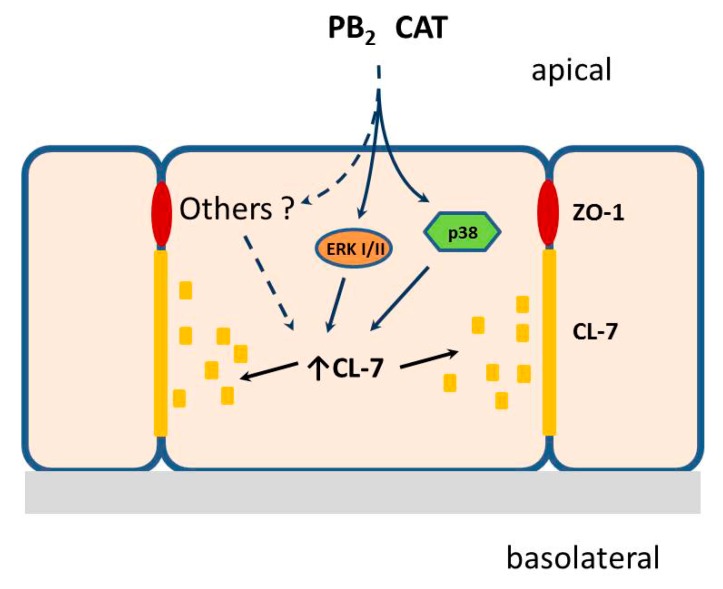
A tentative model to summarize polyphenol-dependent induction of CL-7 in Caco-2/HT29-MTX co-culture system.

An original finding of this investigation is the modulation of TJ proteins by the two polyphenols. Since both CAT and PB_2_ were not able to activate NF-κB, it is likely that the effects of these compounds on TJ proteins were due to alternative mechanisms of transduction. Given the increase in the phosphorylated forms of both ERK1/2 and p38 induced by CAT and PB2 (Figure 4), it is tempting to attribute their effects to MAPK, although the involvement of other pathways cannot be excluded (see the model reported in Figure 6). Moreover, the elucidation of MAPK role needs further investigation, including the effect of suppression of their activity through genetic or pharmacological means. In particular, both CAT and PB_2_ lowered the expression of ZO-1, making it therefore not completely surprising that they do not counteract the fall in the expression of the protein caused by inflammation. On the contrary, occludin expression is increased by both compounds, although only PB_2_ seems to overcome the changes observed in MCM challenged monolayers. The most striking changes concern CL-7 expression and distribution. Both inflammation and the tested polyphenols markedly increased the expression of the protein, but the stimulation observed in cells pre-treated with CAT and PB_2_ was remarkably higher than that detected in cultures stressed by MCM. The two effects are not additive but, rather, inflammation seems to blunt the effect of polyphenols. Moreover, inflammation-dependent dislocation of the CL-7 pattern, highlighted by its increased co-localization with ZO-1, was partially compensated by polyphenols. While the mechanisms underlying these changes have not been investigated, it is tempting to link the increased abundance of CL-7 in the cytoplasm of MCM-perturbed monolayers with the markedly increase in its expression. CL-7 has been found to be significantly downregulated in colorectal cancer samples in a large patient database, and the expression of CL-7 has been associated with mesenchymal to epithelial transition, a phenomenon that counteracts tumorigenesis in colorectal mucosa [59]. Conversely, CL-7 down-regulation has been associated with invasive phenotype in colon cancer [60,61]. Regulation of CL-7 expression has not been completely elucidated thus far, and little information is available on possible nutrient effects, although it is known that neither berberine nor quercetin are able to affect the expression of the protein [62]. Thus, induction of CL-7 by CAT and PB_2_ may represent a novel promising research line to elucidate the potential cancer preventive properties of polyphenols. 

## 5. Conclusions

CAT and PB2 markedly increase the expression of the tight-junctional protein CL-7, without, however, preventing inflammation-dependent impairment of the barrier function of intestinal cell monolayers. Although further studies are required to clarify the involved mechanisms, these results provide novel insights on the biological activities of these major dietary flavan-3-ols at intestinal level. 

## Figures and Tables

**Table 1:** Antibodies used for Western blot and immunofluorescence.

Antibody	Host	Clonality	Dilution	Company
anti-ERK	Rabbit	polyclonal	1:1000 (WB ^1^)	R&D Systems
anti-p-ERK	Rabbit	polyclonal	1:1000 (WB)	R&D Systems
anti-Claudin7	Rabbit	polyclonal	1:1000 (WB); 1:400 (IF ^2^)	Cell Signaling
anti-Occludin	Rabbit	polyclonal	1:1000 (WB)	Thermo Fischer
anti-p38	Rabbit	polyclonal	1:500 (WB)	R&D Systems
anti-p-p38	Rabbit	polyclonal	1:500 (WB)	R&D Systems
anti-p-p65 (NF B)	Rabbit	polyclonal	1:1000 (WB)	Cell Signaling
anti-p65 (NF B)	Rabbit	polyclonal	1:1000 (WB)	Cell Signaling
anti-β-Tubulin	Mouse	monoclonal	1:1000 (WB)	Sigma
Anti-ZO-1	Mouse	monoclonal	1:1000 (WB); 1:400 (IF)	Thermo Fisher

^1^ WB: Western Blot; ^2^ IF: Immunofluorescence.

## Supplementary Materials

The following are available online at https://www.mdpi.com/2072-6643/11/10/2271/s1, Figure S1: Distribution of ZO-1 and CL-7 in Caco-2/HT29-MTX monolayers.
