# Supplementary material for: Catechin and Procyanidin B2 Modulate the Expression of Tight Junction Proteins but Do Not Protect from Inflammation-Induced Changes in Permeability in Human Intestinal Cell Monolayers
Source: Nutrients. 2019 Sep 21;11(10):2271. doi: 10.3390/nu11102271 (PMC6836206; doi:10.3390/nu11102271)
Supplement: Supplementary file 1 [file nutrients-11-02271-s001.pdf]

nutrients

Article

Catechin and Procyanidin B2 Modulate the Expression of Tight Junction Proteins but do not Protect from Inflammation-induced Changes in Permeability in Human Intestinal Cell Monolayers

Massimiliano G. Bianchi [1], Martina Chiu [1], Giuseppe Taurino [1], Furio Brighenti [2], Daniele Del Rio [3,4,5]*, Pedro Mena [2,3] and Ovidio Bussolati [1,4,]*

Supplementary Material

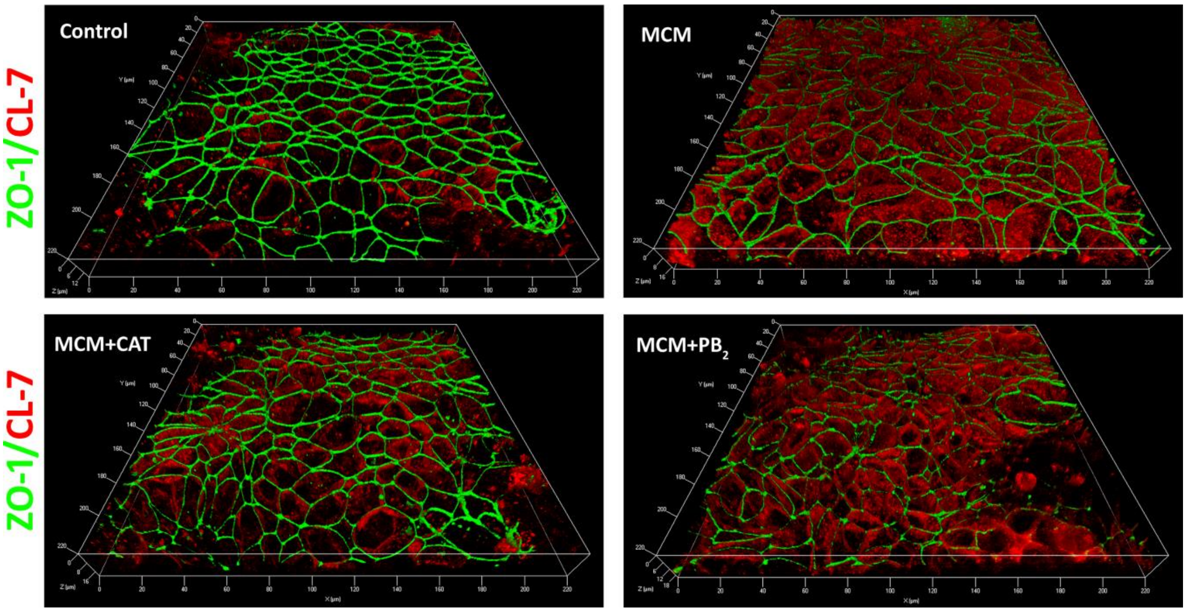

Figure S1. Distribution of ZO-1 and CL-7 in Caco-2/HT29-MTX monolayers. Reconstructions were performed from z-stacks of digital images (minimum 40 confocal sections, z-axis acquisition interval of 0.38 µm) taken during the experiment shown in Figure 3. The images were processed with the Axiovision module inside 4D release 4.5 (Carl Zeiss, Jena, Germany), applying the transparency algorithm. See the legend to Figure 3 for further information. Claudin-7, red; ZO-1, green.
